# Clinical and epidemiological characteristics of pyogenic liver abscess in people 65 years or older versus people under 65: a retrospective study

**DOI:** 10.1186/s12877-017-0545-x

**Published:** 2017-07-21

**Authors:** Jorge Peris, Pablo Bellot, Pablo Roig, Sergio Reus, Sara Carrascosa, Gregorio González-Alcaide, José M. Palazón, José M. Ramos

**Affiliations:** 1 Deparment of Internal Medicine, Hospital Universitario Sant Joan d’Alacant, Sant Joan d’Alacant, Alicante Spain; 2 Department of Clinical Medicine, Universidad Miguel Hernández de Elche, Campus of Sant Joan d’Alacant, Sant Joan d’Alacant, Alicante Spain; 3 Gastroenterology and Hepatology Service, Hospital General Universitario de Alicante, Alicante, Alicante Spain; 4 Unit of Infectious Diseases, Department of Internal Medicine, Hospital General Universitario de Alicante, Alicante, Alicante Spain; 5 Family Medicine Department, Campello Health Centre, El Campello, Alicante Spain; 6 Department of History of Science and Documentation, University of Valencia, Valencia, Valencia Spain; 7 Department of Internal Medicine, Hospital General Universitario de Alicante Alicante, Alicante, Alicante Spain

**Keywords:** Liver abscess, Pyogenic, Epidemiology, Microbiology, Mortality, Aged, Female, *Escherichia coli*

## Abstract

**Background:**

To analyse the clinical, epidemiological, microbiological and prognostic differences of pyogenic liver abscess (PLA) in older (≥ 65 years of age) versus younger patients (< 65 years).

**Methods:**

Multicentre, retrospective cohort study in all patients with PLA admitted to two Spanish hospitals from January 2000 to January 2014. Cases were divided into two age groups (< 65 years and ≥65 years) for comparison of clinical, epidemiological and microbiological characteristics as well as treatment.

**Results:**

Of 98 patients analysed, 40 patients were younger than 65, and 58 were aged 65 or older. Significant associations in the older group were found with female sex (adjusted odds ratio [ORa] 9.0; 95% CI 1.4, 56), non-cryptogenic origin (ORa 14.5; 95% CI 1.6, 129), absence of chronic liver disease (ORa 14; 95% CI 1.3, 155), *Escherichia coli* infection (ORa 7.7; 95% CI 1.03, 58), and incidence of complications (ORa 2.3; 95% CI 1.04, 5.4). Mortality was 8.2% overall, although all deaths occurred in the older group (8/58; 13.8%) (*p* = 0.02).

**Discussion:**

Our results are in consonance with other published studies. Older patients with PLA tend to present more anomalies in the biliary tract (Kai et. al, World J Gastroenterol 18: 2948-295, 2012, Rahimian et. al, Clin Infect Dis 39:1654-9, 2004, Seeto, Medicine (Baltimore) 75:99-113, 1996, Kao et.al, Aliment Pharmacol Ther 36:467-76, 2012, Lai et. al, Gastroenterology 146:129-37, 2014), while younger patients are more often male and present more commonly with previous liver disease (especially related to alcohol) and cryptogenic PLA.

**Conclusion:**

In patients aged 65 or older, PLA was more common in women and in those with a history of biliary disease, and *E. coli* was the most frequent bacterium. Mortality was also higher in the older group.

## Background

Pyogenic liver abscess (PLA) is defined as a collection of pus surrounded by a fibrous layer of tissue in the liver. Reported incidence in the literature ranges from 2 to 86 cases per 100,000 hospital admittances/year [1–4]. However, PLA has doubled in incidence in the most recent decades for several reasons: immunosuppressive treatments in cancer patients and/or transplant recipients, the use of invasive techniques to manage hepatobiliary disease, and greater diagnostic efficacy through imaging techniques [5, 6]. Moreover, some studies point to a possible relationship with colon cancer [7].

Modern diagnostic techniques allow more exact positioning of the PLA, which has facilitated the development of therapeutic techniques such as drainage or aspiration. PLA is associated with high morbidity and mortality; however, these have decreased thanks to early detection and the development of different treatments, ranging from antibiotics to image-guided drainage/aspiration procedures. Despite these advances, studies still estimate a mortality rate of 2 to 31% [3, 4, 8, 9].

The microorganisms most commonly responsible for PLA are *Klebsiella pneumoniae* followed by *Escherichia coli.* In addition, diverse series have described the recent appearance of rarer multi-resistant germs such as *K. pneumoniae* or *E. coli* producers of beta-lactamases or multi-drug resistant *Pseudomonas aeruginosa* [10].

Advanced age is an increasingly important factor for both the prognosis and the severity of numerous pathologies, but few studies describe the clinical features in the elderly compared to younger patients [11, 12]. Thus, this study was conceived to fill that gap in knowledge; it aims to analyse the clinical, epidemiological, microbiological and prognostic differences of PLAs in older (≥ 65 years of age) versus younger patients (< 65 years).

## Methods

Multicentre, retrospective cohort study in all patients with liver abscess admitted to the Hospital de San Juan de Alicante and to the General Hospital of Alicante from January 2000 to January 2014. Among these patients, we performed a systematic search of hospital records in order to select consecutive cases with an abscess corresponding to the International Classification of Diseases (ICD) code ICD-9-CM 572.0. PLA was defined as any abscess found within the liver parenchyma that was of bacterial origin. According to criteria published by Alvarez and colleagues [12], diagnosis was based on clinical features, evidence from imaging studies (e.g. single or multiple space-occupying lesions on liver ultrasonography or abdominal computerised tomography [CT]), blood culture, pus from liver aspirates, surgical findings, and resolution of the lesion(s) after antibacterial chemotherapy. We excluded patients from analysis for the following reasons: fungal abscess, parasitic abscess and amoebic abscess.

We collected data for *demographic variables*: age and sex; *risk factors*: previous chronic liver disease, organ transplant, human immunodeficiency virus, inflammatory intestinal disease (including primary sclerosing colangitis), cancer; *clinical manifestations*: abdominal pain, fever and jaundice; *laboratory parameters*: haemoglobin, total leukocytes, total bilirubin, alkaline phosphatase, gamma glutamil transferase (GGT), C-reactive protein (CRP); *radiological techniques used for diagnosis*: ultrasound, abdominal CT scan, magnetic resonance imaging (MRI); origin and aetiology of PLA; *microbiological results* (from samples of the PLA itself and, when available, blood cultures); and *health care indicators*: treatment (antibiotic alone, antibiotic plus percutaneous aspiration, and antibiotic plus surgical drainage with or without percutaneous aspiration), length of treatment, antibiotic used, complications, length of hospital stay, and mortality.

We classified PLAs as being small (< 4 cm, with no need for drainage), large (4 cm to <10 cm), or giant (≥ 10 cm) (13,14). Chronic liver disease was defined as progressive destruction of the liver parenchyma over a period greater than 6 months leading to fibrosis and cirrhosis, including primary biliary cirrhosis (PBC), primary sclerosing cholangitis, autoimmune hepatitis, drug-induced liver disease or viral chronic hepatitis. The aetiological origin of PLA included: cryptogenic (treating physician could not identify the source of infection despite appropriate investigation), biliary (in patients with gallstones and a clinical picture of cholecystitis/cholangitis), contiguous (in patients with intra-abdominal abscess), portal (in case of a documented abdominal infection such as appendicitis or diverticulitis), arterial (with a documented bacteraemic episode and no history of other intra-abdominal infections) and traumatic (where the abscess was secondary to a previous abdominal surgical intervention) [13].

Cases were divided into two groups based on age (< 65 years and ≥65 years) for statistical analyses of differences. Old age is typically defined as over 65 years based on old UK retirement standards. All continuous data were compiled as mean ± standard deviation; categorical variables are reported as a percentage. We analysed quantitative variables using the student t-test and the Mann-Whitney U test (depending on whether data followed a normal distribution or not), while we used the Chi-squared test for categorical variables. To measure association, we used the odds ratio (OR) or adjusted OR (ORa) and expressed results with 95% confidence intervals (CI). Moreover, we performed multiple logistic regression analyses (simultaneous regression) with age over 65 as an independent variable. Associations showing *p* values of less than 0.05 were re-run in multivariable analyses. We compiled and analysed all data using SPSS software for Windows 22.0 (IBM Inc., New York).

The study was approved by the ethics committee for epidemiologic research at Hospital Universitario Sant Joan d’Alacant (2016/011). Due to the retrospective nature of the study, the need for informed consent was waived.

## Results

Of the 102 patients with PLA, we excluded 4 due to age (paediatric cases in patients under 14). Mean age was 64.5 years (± 15.4; range 19 to 100). Forty patients were younger than 65, and 58 were aged 65 or older. Mean age in the younger group was 49.1 years (±10.0), compared to 79.1 years (±7.3) in the older group.

Table 1 shows the clinical and epidemiological characteristics of patients with PLA. Presence of fever, abdominal pain and jaundice, and history of cancer and colitis, were similar between groups. However, there were significant differences in the following variables in the older group compared to the younger one: female sex (OR 11.9; 95% CI 3.7, 37.7); presence of sepsis (OR 4.3; 95% CI 1.2, 16.0), history of cholelithiasis (OR 4.6; 95% CI 1.6, 13.5), and absence of chronic liver disease (OR 0.2; 95% CI 0.06, 0.7). In the older group, biliary origin was more common (OR 4.9; 95% CI 2.0, 12.1) while cryptogenic origin was less so (OR 0.1, 95% CI 0.05, 0.3).

**Table 1:** Main epidemiological and clinical characteristics of patients aged <65 years and ≥65 years with liver abscess

Variables	Total		Aged <65 years		Aged ≥65 years		Odds ratio (95% CI)	*p*-value
	N	%	N	%	N	%		
Sex								
Male	61	82.2	36	90	25	43.1	1	
Female	37	37.8	4	10	33	56.9	11.9 (3.7, 37.7)	<0.001
Clinical characteristics								
Fever	69	70.4	31	77.5	38	65.5	0.6 (0.2, 1.4)	0.2
Abdominal pain	37	37.9	26	65.0	35	60.3	0.8 (0.4, 1.9)	0.6
Jaundice	27	72.4	10	25.0	17	29.3	1.2 (0.5, 3.1)	0.6
Sepsis	18	18.4	3	7.5	15	25.9	4.3 (1.2, 16)	0.02
Underlying condition								
Cholelithiasis	28	28.6	5	12.5	23	39.7	4.6 (1.6, 13.5)	0.003
Chronic liver disease ^a^	15	15.3	11	27.5	4	6.9	0.2 (0.06, 0.7)	0.005
Neoplasm ^c^	13	13.3	3	7.5	10	17.2	2.6 (0.7, 10.0)	0.2
Transplant recipient^b^	5	5.1	5	12.5	0	0	N.A	0.01
Inflammatory bowel disease	5	5.1	4	10.0	1	1.7	0.2 (0.01, 1.5)	0.2
Origin								
Biliary	43	43.9	9	22.5	34	58.6	4.9 (2.0, 12.1)	<0.001
Cryptogenic	40	40.8	27	67.5	13	22.4	0.1 (0.05, 0.3)	<0.001
Contiguous	6	6.1	2	5.0	4	6.9	1.4 (0.2, 8.0)	0.7
Traumatic	5	5.1	2	5.0	3	5.2	1.0 (0.2, 6.5)	1
Portal	3	3.1	0	0	3	5.2	NA	0.3
Arterial	1	1.0	0	0	1	1.7	NA	1

*CI* confidence intervals, *NA* not applicable

^a^ Chronic hepatopathy: alcoholic hepathopathy (*n* = 7), hepatitis C virus (*n* = 4); hepatic steatosis (*n* = 3); and cirrohosis (*n* = 1)

^b^ Transplant recipient*: 3 liver and 2 kidney

^c^ Type of neoplasm: hepatocarcinoma (*n* = 4), adenocarcinoma of pancreas (*n* = 3), neoplasms of kidney (*n* = 2), cholangiocarcinoma (*n* = 1), hepatic metastasis (*n* = 1), adenocarcinoma of colon (*n* = 1), adenocarcinoma of breast (*n* = 1)

The most frequent combination of imaging techniques used was abdominal ultrasound plus CT scan (48%), followed by ultrasound plus CT plus MRI (18%). Table 2 shows the comparison of techniques used in both groups. Mean PLA diameter in the older group was smaller (4.2 ± 3.0 cm vs. 5.6 ± 3.6 cm; *p* = 0.04).

**Table 2:** Imaging procedures of patients aged <65 years and ≥65 years with liver abscess

	Total		Aged <65 years		Aged ≥65 years		Odds ratio (CI 95%)	*p*-value
	N	%	N	%	N	%		
Diagnostic imaging procedure								
CT scan	83	84.7	38	95.0	45	77.6	0.2 (0.04, 0.8)	0.02
Ultrasound	80	81.6	30	75.0	50	86.2	2.1 (0.7, 5.8)	0.2
MRI	29	29.6	14	35.0	15	25.9	0.6 (0.3, 1.6)	0.3
Single or multiple								
Single	54	55.1	34	58.6	20	50.0	1	0.4
Multiple	44	44.9	20	45.5	24	41.4	0.7 (0.3, 1.6)	
Size category								
< 4 cm	46	46.9	15	37.5	31	53.4	1.9 (0.8, 4.3)	<0.15
4–9.9 cm	42	42.9	20	50.0	22	37.9	0.6 (0.7, 1.4)	0.235
≥ 10 cm	10	10.2	5	12.5	5	8.6	0.6 (0.2, 2.4)	0.736
	mean	SD	mean	SD	mean	SD		
Side diameter (cm)	4.8	3.3	5.6	3.6	4.2	3.0	0.9 (0.8, 0.99)	0.04
Other imaging findings								
Gallstones	28	28.6	5	12.5	23	39.7	4.6 (1.6, 13.5)	0.003
Gas formation	6	6.1	2	5.0	4	6.9	1.4 (0.2, 8.0)	0.7
Hepatocarcinoma	4	4.1	1	2.5	3	5.2	3.2 (0.5, 6.0)	0.9
Neoplasm of pancreas	3	3.1	0	0	3	3.1	NA	1
Neoplasm of kidney	2	2.0	0	0	2	3.2	NA	1
Cholangiocarcinoma	1	1.0	0	0	1	1.7	NA	1
Hepatic metastasis	1	1.0	1	2.5	0	0	NA	1

*CI* confidence intervals, *SD* standard deviation

*CT scan* computerised tomography scan, *MRI* magnetic resonance imaging

Table 3 compares laboratory data between groups. There were no significant differences in leukocyte counts, haemoglobin, alkaline phosphatase, total bilirubin or CRP. In contrast, there was a difference in GGT levels, which were higher in the older group.

**Table 3:** Laboratory data of patients aged <65 years and ≥65 years with liver abscess

Variables	Missing values	Total		Aged <65 years		Aged ≥65 years		*p*-value
		Mean	SD	Mean	SD	Mean	SD	
Leukocyte ≥12.0 × 10^9^/L	0:4	14.3	7.0	13.7	4.9	14.8	8.2	0.4
Haemoglobin (g/L)	1:4	12–0	2.2	12.0	2.1	12.3	2.3	0.1
Alkaline phosphatase (mg/dL)	8:12	208	160	179	149	228	168	0.3
Gamma glutamyl transferase (IU/dL),	18:10	338	405	219	189	437	503	0.04
Total bilirubin (mg/dL),	1:8	1.7	1.7	1.8	1.9	1.6	1.6	0.7
C reactive protein (mg/dL)	5:9	18.1	11.8	19.7	12.7	17.0	10.9	0.3

*SD* standard deviation

The main microbiological diagnostic procedures were cultures of PLA tissue samples and blood cultures, which were both performed in 42.9% of the cases, followed by blood culture alone. Cultures were positive in 67.7% of the PLA tissue samples and 33.9% of the blood cultures. The main microorganism isolated as the cause of the PLA was *E. coli* (16.3%), followed by *Streptococcus anginosus* group (13.3%) (Table 4). *E. coli* was significantly more frequent in the older group (OR 6.0; 95% CI 1.3, 28.4). Four gram-negative bacteria were identified as producers of beta-lactamases (two *E. coli* and two *K. pneumonia*): two in the older group (3.4%) and two in the younger group (5.0%).

**Table 4:** Microbial diagnostic procedure and microbiological diagnosis of patients aged <65 years and ≥65 years with liver abscess

Variables	Total		Aged <65 years		Aged ≥65 years		Odds ratio (CI 95%)	*p*-value
	N	%	N	%	N	%		
Microbial diagnostic procedure								
No culture	19	19.4	5	12.5	14	24.1	2.2 (0.7, 6.8)	0.1
Culture of abscess	20	20.4	7	17.5	13	22.4	1.3 (0.5, 3.8)	0.5
Blood culture	17	17.3	6	15.0	11	19.0	1.3 (0.4, 3.9)	0.6
Culture of abscess + blood culture	42	42.9	22	55.0	20	34.5	0.4 (0.2, 0.98)	0.04
Results								
Positive culture of abscess	44/65	67.7	21/30	70	23/35	65.7	0.8 (0.3, 2.3)	0.7
Positive blood culture	20/59	33.9	9/28	32.1	11/31	35.5	1.1 (0.3, 3.4)	0.8
Microorganisms								
*Escherichia coli*	16	16.3	2	5.0	14	24.1	6.0 (1.3, 28.4)	0.01
*Streptococcus anginosus* group ^a^	13	13.3	7	17.5	6	10.3	0.5 (0.7, 1.7)	0.2
*Klebsiella* spp*.* ^b^	11	11.2	7	17.5	4	6.9	0.3 (0.09, 1.2)	0.1
*Enterococcus* spp*.*	5	5.1	1	2.5	4	6.9	2.9 (0.3, 26.8)	0.3
*Streptococcus mitis*	4	4.1	3	7.5	1	1.7	0.2 (0.02, 2.1)	0.2
*Porphyromonas* spp*.*	3	3.1	1	2.5	2	3.4	1.4 (0.1, 15.9)	0.8
*Pseudomonas aeruginosa*	3	3.1	2	5.0	1	1.7	0.3 (0.03, 3.8)	0.4
*Citrobacter* spp*.*	2	2.0	0	0	2	3.4	NA	0.2
*Enterobacter cloacae*	1	1.0	0	0	1	1.7	NA	1
*Fusobacterium* spp*.*	1	1.0	1	2.5	1	1.7	NA	1
*Eikenella corrodens*	1	1.0	0	0	1	1.7	NA	1
*Candida albicans*	1	1.0	0	0	1	1.7	NA	1
*Magnusiomyces capitatus*	1	1.0	0	0	1	1.7	NA	1
Total patients with positive cultures	54	55.1	23	57.5	31	53.4	0.8 (0.4, 1.9)	0.7
Polymicrobial infections	7	7.1	3	7.5	4	6.9	0.9 (0.2, 4.3)	0.9

*CI* confidence intervals, *NA* not applicable

^a^ *Streptococcus anginosus* group include *S. constellatus, S. milleri, S. intermedius and S. anginosus*

^b^ *Klebsiella* spp. includes: *K. pneumoniae and K. oxytoca*

Most patients received antibiotics plus percutaneous drainage (61.2%) followed by antibiotic alone (31.6%) and antibiotic plus surgical drainage (7.1%); there was no significant difference between groups (Table 5). The main antibiotics used were penicillin/beta lactamase inhibitors (31.6%) followed by metronidazole (26.5%) and carbapenems (23.5%); there was no significant difference between groups.

**Table 5:** Treatment, complications and outcomes of patients aged <65 years and ≥65 years with liver abscess

	Total		Aged <65 years		Aged ≥65 years		Odds ratio (CI 95%)	*p*-value
	N	%	N	%	N	%		
Treatment								
Antibiotic alone	31	31.6	11	27.5	20	34.5	1.4 (0.6, 3.3)	0.5
Antibiotic + percutaneous aspiration	60	61.2	28	70	32	55.2	0.5 (0.2, 1.2)	0.1
Antibiotic + surgical drainage ± percutaneous aspiration	7	7.1	1	2.5	6	10.3	4.5 (0.5, 38.9)	0.1
Antibiotics used^a^								
Penicillins/beta-lactamase inhibitors	31	31.6	16	40	15	25.9	0.5 (0.2, 1,2)	0.1
Metronidazole	25	26.5	14	35.0	12	20.7	4.5 (0.2, 1.2)	0.1
Carbapenems	23	23.5	9	22.5	14	24.1	1,1 (0.4, 2.8)	0.8
Fluoroquinolones	20	20.4	7	17.5	13	22.4	1.4 (0.5, 3.8)	0.5
Cephalosporins	15	25.5	8	20.0	17	29,3	1.7 (0.6, 4.3)	0.3
Complications								
Any complication	54	55.1	17	42.5	37	68.5	2.3 (1.04, 5.4)	0.04
Severe complications	25	25.5	5	12.5	20	34.5	3.7 (1.2, 10.8)	0.01
Type of complications								
Septic shock	14	14.3	2	5.0	12	20.7	4.9 (1.04, 23)	0.03
Pleural effusion	14	14.3	6	15.0	8	13.8	0.9 (0.3, 2.8)	0.9
Acute renal failure	8	8.2	1	2.5	7	12.1	5.3 (0.6, 45.3)	0.09
Cardiac complications	8	8.2	1	2.5	7	12.1	5.3 (0.6, 45)	0.09
Thrombosis	6	6.1	4	10.0	2	2.0	0.3 (0.05, 1.8)	0.2
Biliary-pancreatic complication	5	5.1	2	5.0	3	5.1	2.1 (0.5, 11)	0.8
Infectious complications	4	4.1	1	2.5	3	5.2	2.1 (0.2, 21, 2)	0.5
Peritonitis	1	1.0	0	0	1	1.7	NA	1
Abdominal haematoma	1	1.0	0	0	1	1.7	NA	1
Outcome								
Death	8	8.2	0	0	8	13.8	NA	0.02
	mean	SD	mean	SD	mean	SD		
Admission days	19.3	12.8	19.5	11.9	18.9	13.5	NA	0.728

*CI* confidence intervals, *NA* not applicable, *SD* standard deviation

^a^Other antibiotics: amynoglucosides (*n* = 4), glycopetide (*n* = 3), clindamicin (*n* = 1)

Table 6 describes the treatment used according to PLA size. Small PLAs were treated mainly with antibiotic alone (*p* < 0.001), while both large PLAs were treated with antibiotic plus percutaneous aspiration (*p* < 0.001). These seven patients who were treated with surgical drainage received it due to: *cholecystitis* and biliary complication (*n* = 4), neoplasm (*n* = 2) and peritonitis with continuous fever (*n* = 1).

**Table 6:** Treatment and mortality according to size of liver abscess

	Size category						
	< 4 cm (*n* = 46)		4–9.9 cm (*n* = 42)		≥ 10 cm (*n* = 10)		*p*-value
	N	%	N	%	N	%	
Treatment							
Antibiotic alone	24	52.2	7	16.7	0	0	<0.001
Antibiotic + percutaneous aspiration	19	41.3	32	76.2	9	90	0.001
Antibiotic + surgical drainage^a^	3	6.5	3	7.1	1	10	0.9
Mortality	4	8.7	4	9.5	0	0	0.9

^a^ ± percutaneous aspiration

Table 5 compares the adverse events encountered in the 55.1% of patients who experienced them (25.5% of which were severe). The incidence of all types of complications and severe complication was higher in the older group (OR 2.3; 95% CI 1.04, 5.4 and OR: 3.7; 95% CI 1.2, 10.8, respectively) compared to the younger one. The most frequent complications were pleural effusion (14.3%) and septic shock (14.3%), the latter of which was more common in the ≥65 group (OR 4.9; 95% CI: 1.04, 23.0). Mortality was 8.2% overall, although all deaths occurred in the older group (13.8%) (*p* = 0.02). On the other hand, the length of hospital stay was similar between groups.

Finally, Table 7 shows the results of the multivariable analysis in patients aged ≥65 years with PLA. Significant associations were found with female sex (ORa 9.0; 95% CI 1.4, 56.1), non-cryptogenic origin (ORa 14.5; 95% CI 1.6, 129.0), absence of chronic liver disease (ORa 14.0; 95% CI 1.3, 155.1), and *E. coli* infection (ORa 7.7; 95% CI 1.03, 58.0).

**Table 7:** Multivariable analysis in patients ≥65 years with liver abscess

	Odds ratio adjusted (CI 95%)	*p*-value
Sex, female	9.0 (1.4, 56.0)	0.02
Source of origin: non-cryptogenic	14.5 (1.6, 129.0)	0.02
Underlying condition: not chronic liver disease	14 (1.3, 15.05)	0.03
Infection due to *E. coli*	7.7 (1.03, 58.0)	0.05
Side diameter	0.8 (0.08, 1.9)	0.1
Source of origin: biliary	5.1 (0.5, 58.3)	0.2
Any complications	2.6 (0.5, 12.5)	0.2
Septic shock	24 (0.2, 3568)	0.2
Underlying condition: cholelithiasis	2.6 (0.4, 14.1)	0.3
Clinical presentation, sepsis	0.2 (0.01, 3.3)	0.3
Specific severe complications	1.3 (0.08, 20.1)	0.8
Underlying condition: transplant recipient	NA	0.9
Death	NA	0.9

*CI* confidence intervals

In total, eight patients (8.2%) died due to PLA. The leukocyte counts in the patients who died were significantly higher than in those whose clinical evolution was favourable (20.1 ± 11.1 × 10^6^/L vs. 13.9 ± 6.6 × 10^6^/L) (*p* = 0.01). All of the patients who died experienced sepsis, compared to 11.1% of those who survived, and 87.8% presented with septic shock, compared to 7.8% of the survivors (*p* < 0.001). The rest of the variables explored were not related to mortality. In the multivariable analysis, no association was observed with mortality.

## Discussion

Although overall incidence of PLA remained stable throughout the study period, its distribution between age groups showed a clear variation. In the earliest years, the most affected age groups corresponded to people aged 30–49 years, while more recent periods show a higher incidence among patients aged 50–69 [14–17]. This trend can be attributed to the decreasing incidence of abscesses related to acute appendicitis as an aetiopathogenic factor (more common among younger patients) and the greater incidence of neoplasms and complex biliary diseases observed at more advanced ages [1, 2, 14–17].

In this study, we describe our experience in our area within the context of existing literature. We have found some significant differences in the <65 and ≥65 age groups. There were more women in the older age group, and older patients also more commonly had a history of biliary pathology and no chronic liver disease, with a greater likelihood of *E. coli* infection.

Our results are in consonance with other published studies. Older patients with PLA tend to present more anomalies in the biliary tract [7, 8, 18, 19, 20], while younger patients are more often male and present more commonly with previous liver disease (especially related to alcohol) and cryptogenic PLA [19].

With regard to epidemiologic characteristics, our findings in elderly patients were also consistent with the literature, with a predominance of women in the older group and men in the younger group [8, 21, 22]. This may be due to the different life expectancies between sexes as well as the greater prevalence of chronic liver disease among younger patients in our study. In contrast, the presence of cholelithiasis was more pronounced in the older group.

In the univariate analysis only, neoplasms (particularly hepatocarcinoma) were significantly more prevalent in the older group, whereas inflammatory bowel disease was significantly less prevalent in the younger group (*p* > 0.05). In other studies, older patients with PLA tend to present more underlying malignant diseases [7, 8, 19, 20]. Moreover, transplant recipients were only in the younger group, as advanced age is an exclusion criterion for liver transplant waitlists.

The advantage of CT scans over ultrasounds resides in the detection of small (≤ 0.05 cm diameter) deposits and the possibility of correctly exploring the superior subsegment of the posterior segment of the liver [23–25]. Moreover, the CT can provide information about the existence of PLA-associated disease outside the liver, such as appendicitis, diverticulitis, cancer, etc. In light of these advantages, many authors consider the CT to be the best initial diagnostic procedure in patients with suspicion of PLA [23].

Recently, MRI has been incorporated into the diagnostic toolkit for the study of abdominal pathologies, rivalling the CT in the detection of PLA. A theoretical advantage of the MRI over the CT is that it provides better information about the hepatic venous anatomy; however, the relationship between an abscess and the hepatic veins can be seen by ultrasound [25]. With regard to the use of diagnostic imaging techniques, we did not find statistically significant differences between groups. The ultrasound and CT are used widely, to a much greater extent than the MRI. In any case, age does not appear to be a significant factor for physicians when deciding which radiologic method to use. Consistent with other studies, PLA alone was the most frequent form of presentation in both of our groups. On the other hand, the maximum diameter was greater (and showed statistical significance) in the younger group.

With regard to the laboratory findings at the time of the consult, we did not observe significant differences in the variables collected, including bilirubin or leukocyte values. These data are consistent with those reported elsewhere [1, 4, 5]. We only found a significant difference in the higher value of GGT among the older group, probably due to the greater biliary aetiology of liver abscesses in this study.

The main microbiological technique was the culture of PLA tissue, which was usually accompanied by blood culture. We did not observe significant differences between groups, either in the technique used or the positivity found. Traditional series show *S. aureus* to be the principal germ isolated in hepatic cultures, but *E. coli* is also a common cause of PLA [25]. *K. pneumoniae* PLA have been widely described, particularly in southeast Asian countries [26, 27]. In in our series, *K. pneumoniae* was not the most common microorganism, but rather *E. coli* followed by the *S. anginosus* group; *K. pneumoniae* ranked third. The differences observed in our study may be because we did not perform enough blood cultures or obtain sufficient pus for cultures, or possibly because antibiotics were administered before cultures were obtained. In this case, cultures may not test positive the first time, and isolating the microorganism may require multiple attempts [28]. In a recent comparative study of PLA in 264 patients with *K. pneumonia* and 24 patients with *E. coli,* the latter bacterium affected older patients and was more commonly associated with hyperbilirubinemia, increased GGT and gallstones [13]. Likewise, in our study *E. coli* and gallstones were significantly more common in patients over 65.

Today, surgical treatment of PLA has been relegated in favour of the current frontline treatments of antibiotics and aspiration/drainage of the PLA [29, 30, 31]. Moreover, laparoscopic drainage of PLA has also recently been developed as an option for surgical drainage of liver abscess [32]. Our series also reflected this situation; the first treatment was antibiotics and percutaneous aspiration/drainage of PLA, followed by antibiotic alone. Few patients received antibiotics plus surgical treatment, and only one underwent laparoscopic drainage. There were no differences in treatment of PLA between age groups, nor was type of treatment associated with mortality. This observation points to both the appropriateness of the treatment and to the fact that therapeutic efforts are not rationed in older patients.

The use of antibiotics was variable in our study, as in this clinical practice, each attending physician decided the treatment, its duration and the time for switching from intravenous to oral administration. This is a limitation of all retrospective studies, including ours. In intra-abdominal infections, it is important remember to appropriately prescribe antibiotics – and to limit the length of treatment – in order to avert antibiotic resistance [33]. Moreover in our study, the beginning of the antibiotic treatment was not recorded; however, when PLA (or other intra-abdominal infections) is suspected, quickly starting a course of antibiotics reduces mortality [34].

The appearance of complications, especially septic shock, was more common in patients aged ≥65 years. This finding can be explained by the elevated comorbidity in older patients (neoplasm, diabetes mellitus, etc.) [35]. In our study the mortality was 8.2% overall, although all deaths occurred in patients aged ≥65 years (about 14% of cases in this age group). The mortality in these cases was not really due to age, but rather comorbidities and PLA complications in patients with comorbidities; these factors were more important than age alone. Recently, Martínez-Cecilia et al. reported that octogenarians have comparable outcomes to younger people in colorectal liver metastases when treated with laparoscopic liver resection [36]. Thus, comorbidity and complications are more important prognostic factors than age.

Our study has the inherent limitations of all retrospective studies, particularly the risk of information bias, as it was not always possible to extract all necessary information about the cases.

## Conclusions

In conclusion, in our study, there were more women in the ≥65 age group, and older patients also more commonly had a history of biliary pathology and no chronic liver disease, with a greater likelihood of *E. coli* infection. Death and complications, especially sepsis, were more frequent in older patients, justifying increased efforts for early detection and prompt treatment in this group.

## Abbreviations

CI: Confidence intervals
CRP: C-reactive protein
CT: Computerized tomography
GGT: Gamma glutamil transferase
ICD: International Classification of Diseases
MRI: Magnetic resonance imaging
OR: Odds ratio
ORa: Adjusted OR
PLA: Pyogenic liver abscess
